# Current status of veterinary public health activities in Bangladesh and its future plans

**DOI:** 10.1186/s12917-019-1879-8

**Published:** 2019-05-22

**Authors:** M. Minara Khatun, M. Ariful Islam, M. Mufizur Rahman

**Affiliations:** Department of Microbiology and Hygiene, Faculty of Veterinary Science, Bangladesh Agricultural University, Mymensingh, 2202 Bangladesh

**Keywords:** Veterinary public health, Present status, Constraints, Future plans, Bangladesh

## Abstract

**Background:**

Veterinary Public Health (VPH) is a major part of public health in which human health and well-being are the central tasks. In recent years the VPH is gaining increasing importance because immense changes have occurred in animal production processes and agricultural structures. The aim of this paper is to describe the current VPH activities in Bangladesh, its major constraints and future activities plan to ensure safe food production as well as protect the environment and public health.

**Main text:**

VPH concerns all areas of food production and safety, zoonosis control, environmental protection and animal welfare. In Bangladesh, the VPH unit was established in 1984 by the Directorate of Livestock Services (DLS) for zoonosis control and production of wholesome food of animal origin. Zoonoses are the core domain of VPH. Bangladesh is facing the emergence of zoonotic diseases including anthrax, tuberculosis, brucellosis, salmonellosis, campylobacteriosis, *E. coli* infections, avian influenza, rabies, nipah and dengue virus infections.. Multi-drug resistance bacteria are emerging due to indiscriminate uses of antibiotics in livestock and poultry industries. Lack of proper slaughter houses, antemortem and postmortem inspections of carcasses by qualified veterinarians contributes greatly to unwholesome meat production. The VPH unit has a significant role to play to ensure better public health. However, there are many constraints that affect the VPH services. Absence of VPH services at all administrative level, inadequate budget, lack of qualified personnel, poor lab facilities, absence of legal framework, and the lack of coordination with health department are the major constraints.

**Conclusions:**

The spectrum of VPH issues in Bangladesh is very large. Therefore it is important to carefully set priorities in order to ensure effective and efficient VPH services. Establishment of VPH units at all levels, effective surveillance for zoonotic diseases, institution of legal framework to define role of VPH services, creation of public health awareness, collaborative works with health departments, improving laboratory facilities and training programs for the veterinarian are keys to ensure better VPH services in Bangladesh.

## Background

The term “Veterinary public health,” according to Schwabe [1] was introduced after World War II by public health administrators in the US Public Health Service to designate those areas of public health in which veterinary medicine shares particular interests. In the 1999 joint FAO/WHO expert committee agreed on the following definition of VPH: “VPH is the sum of all contributions to the physical, mental and social well-being of humans through understanding and application of veterinary science”. This definition involves attention to the risks at the level of both production and consumption in food of animal origin. The WHO [2] describes VPH as “a component of public health activities devoted to the application of veterinary skills, knowledge and resources to the protection and improvement of human health.”

VPH is a major part of public health where human health and human well-being are the central tasks [3]. The new consensus definition of VPH deals not only the physical well-being of humans, but also the role of animals for the mental and social well-being of humans. The importance of VPH in recent years is getting increasing attention because immense changes have occurred in animal production processes and agricultural structures. The trade in animals and products of animal origin is increasing due to the loss of border controls within countries. This trade demands new and elaborate surveillance strategies. The active surveillance of animal diseases, their distribution routes and control of emerging and re-emerging diseases is one of the major tasks of VPH [4]. It includes the analysis of the risk for humans and taking effective interventional steps for the protection of human health.

The scope of VPH is clearly multi-disciplinary, involving veterinarians, health professionals and scientists as well as paraprofessionals who treat, control or prevent diseases of animal origin [5]. This discipline of veterinary medicine is global in its potential impact and contribution as is public health. The needs and opportunities for veterinarians are expanding in a number of organizations including public agencies working on human and animal health [6].

The horizon of VPH is expanding rapidly and based upon the present veterinary skills, knowledge and resources; it has been recognized now as an essential field in public health activities all over the world. The chief aim is to protect and improve human health and welfare. In the past this discipline was evolved only to deal with three different issues: combating animal diseases, performing meat inspection and control of zoonoses [7]. At the present time VPH encompasses a wide variety of professional areas linking the three elements such as control of zoonoses, control of food borne pathogens and chemical residues and conserving the environment [7, 8]. Additionally animal welfare is also managed by the VPH service in the United States [9].

The main function of VPH is to control and prevent zoonotic diseases that can be transferred from animals to humans [10]. Transmission of zoonotic diseases occurs either through consuming contaminated foodstuffs or through contact with infected animals. Zoonoses consist of a wide range of diseases including anthrax, brucellosis, bovine tuberculosis, hydatid disease, echinococcosis, trichinellosis, rabies, highly pathogenic avian influenza, Nipah/Hendra disease and bovine spongiform encephalopathy [11]. A risk based program should be carried out in preventing or controlling the transmission of zoonoses. In order to implement an effective zoonoses control program attention should be paid to ecological, cultural, social and ethical aspects [11].

The protection of foodstuffs of animal origin for human consumption, to guarantee their safety and nutritional quality and to prevent disease transmitted in this way is the main responsibilities of the VPH [10, 12]. Safe food plays an important role on the quality of life, whether domestically produced and consumed, imported or exported. Increased presence of additives, pesticides, antibiotics and hormones in foods of animal origin are other emerging VPH problems [13]. Intentional contamination of food has become a serious threat to consumers. Over the last decades, the food chain approach has been recognized as an important step forward to ensure food safety from production up to consumption [11]. This approach requires the commitment of all players in the food chain, involving producers, traders, processors, distributors, competent authorities as well as consumers. VPH service has the responsibility to conduct ante and postmortem inspection of carcasses to ensure wholesome meat for human consumption [14].

The quality of animal feed is important for the production of safe food for human consumption [11].

A core function of the VPH service is to protect the environment [15]. Air pollution, water pollution and soil pollution can cause a direct risk to human *health*. Environmental pollutants arise from animal waste products including chemicals that may be used during production. In addition practicing vets will also produce potential environmental contaminants in the form of used needles, syringes, animal tissue and other clinical waste [16]. All of these materials have to be dealt with in a safe and controlled way to conserve the environment.

Monitoring animal welfare is one of the important functions of the public health veterinarian. Public health veterinarians have the scientific and medical training to ensure animal welfare that enables them to judge the welfare of their patients and clients [17].

## Main text

### VPH activities in Bangladesh and its major constraints

The VPH unit was established in 1985 at Mohakhali, Dhaka under the Research, Training and Evaluation (RTE) section of DLS. Its mandate is to control of zoonotic diseases and ensure safe and wholesome food of animal origin through sanitary inspection of meat and milk and monitoring antibiotic, hormone and chemical residues at all stages of production and up to final consumption [11].

### Control of zoonotic diseases

The prevention and control of zoonotic diseases is the core domain of VPH in Bangladesh. The main bacterial zoonotic diseases in Bangladesh are anthrax, tuberculosis, brucellosis, salmonellosis, campylobacteriosis and leptospirosis. The common viral zoonotic diseases are avian influenza, rabies, nipah virus infection, dengue fever, Japanese encephalitis and rota virus infection. The parasitic zoonotic diseases included cysticercosis and echinococcosis. Toxoplamosis, amebiasis, giardiasis, leishmaniasis, cryptosporidiosis are protozoal zoonotic diseases in Bangladesh. Among the zoonotic diseases rabies, nipah virus infection degue fever and anthrax should get the top priority since these disease causes significant human morbidity and mortality every year in Bangladesh [18]. Poverty is recognized as being a major risk factor for zoonoses and food borne illnesses in both rural and urban consumers [19]. The VPH unit is tasked to maintain relations with the Health Department and the Public Health Institute for implementing surveillance and control program for zoonoses and food safety activities. However, the VPH unit of the RTE section of the DLS virtually lacking effective collaborative program together with the Health Department and the National Institute of Preventive and Social Medicine (NIPSOM). Moreover this unit is under budgeted and does not have sufficient skilled manpower. Lack enforcement of policies, laws, regulations and standards are important constraints in prevention and control of zoonotic diseases.

### Sanitary inspection of meat and milk

Meat inspection and abattoir management are almost absent in rural areas. People in the rural population do not have the opportunity to buy safe and hygienically produced meats. In major cities, slaughter of small ruminants takes place at the markets, while cattle are usually processed in slaughterhouses or slabs that are managed by city corporations. Municipalities and city corporations are entrusted to carry out meat inspection in their jurisdiction usually with government veterinarians. The structures of the most of slaughter houses are poor and slaughter takes place on the ground in quite unhygienic circumstances. Effluent handing is often appalling and is a public health hazard in itself. In most instances there is no ante-mortem and post-mortem inspection by a veterinarian. With respect to foods of animal origin like milk, sweet meats etc., the sanitary inspectors of health department are the authority of inspection and examination [19]. The sanitary inspectors are not properly trained and not supervised by competent veterinarians. Milk quality control is also performed by the private sector, e.g. corporations marketing milk. The VPH unit has not established links with them.

### Monitoring antibiotic, hormone and chemical residues in food of animal origin

Indiscriminate use of antibiotics, especially in the poultry industry is another public health issue. Antibiotics, used as growth promoters, may contribute to the buildup of bacterial resistance to antibiotics [20]. Consumers should be assured that residual levels of antibiotics, pesticides and hormones in food of animal origin are below maximum permitted levels. The VPH unit has no facility to determine these residues in foods. Legislation and enforcement systems to safeguard food for human consumption are lacking.

### Measures need to be adopted to address the public health issues in Bangladesh

VPH, food hygiene and zoonoses control in Bangladesh are identified as weak areas that need to be improved. It is important to carefully set priorities in the current VPH service for effectively and efficiently address the public health issues that are listed below:

### Surveillance of zoonotic disease

Effective surveillance of zoonotic disease needs to be established. It is important to develop surveillance methodology and laboratory facilities. Develop systems to assure that the information collected should be incorporated in the animal health information system. It will be essential to establish risk-based surveillance programs that involve community participation. These are important to perform risk analysis, for which capacity will have to be established at the national level. Single zoonotic/food-borne disease risk analysis and burden assessment can be used as a catalyst for strengthening VPH structures and functions. Joint training of veterinarians and physicians is recommended to better identify local priorities as well as community knowledge, attitudes, and practices. Upazilla Veterinary officer should be responsible for teaching awareness among farmers and consumers, and be responsible for monitoring zoonotic diseases and food safety (slaughterhouses, meat inspection, drug residue, hormone, feed additives testing etc.) and maintaining a liaison with Upazilla health officer and at national level with the VPH department.

### Development of slaughter facilities and ensuring routine meat inspection

To prevent environmental pollution, slaughter of animals in any place other than approved for the purpose should be stopped. Internationally acceptable National Standards for Sanitation and Meat Hygiene should be introduced and enforced in all abattoirs, slaughter houses and plants [21–23]. These facilities should be inspected yearly jointly by the VPH unit of DLS and Bangladesh food safety authority. Establish meat inspection services as a delegated responsibility of the central veterinary authority. Provision of effective training course for meat inspectors and veterinarians are essential for effective meat inspection [24].

### Establishment of institutional legal framework

To establish an efficient, effective and sustainable VPH services, it will require an institutional and legal framework that defines the role of VPH service providers and establishes the functions, responsibilities and control of public, private veterinarians (including the ones working for Non-Government Organizations (NGOs) and private companies and veterinary auxiliaries in the provision of VPH services. This framework should also provide guidelines on the roles, interrelationships and regulations required to link paraprofessionals, private operators and NGOs to the official VPH services [25].

### Creation of public awareness

Raising awareness about various aspect of public health problems related to food of animal origin at the community level is key for successful implementation of VPH program. Public awareness includes: creating awareness on the risks associated with bad eating habits; risks of drug residues in food and importance of meat inspection.

### Monitoring of antibiotic and chemical residues

Through adoption of good agricultural practices, including good animal husbandry and good veterinary practices, it is possible to reduce the necessity for antimicrobials in animal production. Implementation of these activities should be conducted as per the principles laid down in WHO and Office International Epizooties (OIE) guidelines [26]. This will require capacity building, networking and coordination to facilitate implementation. The VPH unit will have to establish the capacity to perform residue testing.

### Building implementation capacity

Extend and incorporate VPH activities into existing primary human and animal health care services. The VPH section should be strengthened with adequate laboratory facilities and trained veterinarians. This section should establish collaborative training and research programs with NIPSOM. At Upazilla level facilitate the organization of a VPH stakeholder association that represent veterinarians, public health physicians, paraprofessionals, livestock producers, consumers and other relevant groups that may play a role in VPH service delivery. Provide continuing education for technical staff including human and animal health workers and post-graduate training for veterinarians as well as health professionals.

## Conclusions

The scope of VPH issues in Bangladesh is very large and clearly multi-disciplinary. Therefore it will be important to carefully set priorities in order to be effective and efficiently use the scarce available resources. A body of legal framework is required to establish an effective, efficient and sustainable VPH services that defines the role of VPH service providers and establishes the functions, responsibilities and control of public, private veterinarians (including the ones working for NGOs and private companies) and veterinary auxiliaries in the provision of VPH services. Veterinarians are in an ideal position to address the concerns of public health. The involvement of veterinary expertise at the policy level is necessary to ensure adequate planning, design, implementation and supervision of VPH programs and to ensure appropriate integration and collaboration with human health programs. The veterinary professionals contribute to improvement of human health by advancing biomedical and comparative medical research, preventing and addressing zoonotic diseases and enhancing environmental and ecosystem health. More research should be adopted related to public health as well as epidemiology of zoonotic diseases so that upcoming Veterinarians might take the challenges of public health concern. Therefore, it is highly recommended that the VPH section should be strengthened with adequate laboratory facilities and trained veterinarians.

## Abbreviations

DLS: Directorate of Livestock Services
FAO: Food and Agricultural Organization
NGOs: Non-Government Organizations
NIPSOM: National Institute of Preventive and Social Medicine
OIE: Office International Epizooties
RTE: Research, Training and Evaluation
VPH: Veterinary Public Health
WHO: World Health Organization
